# Virtual agent-mediated appraisal training: a single case series among Dutch firefighters

**DOI:** 10.1080/20008198.2017.1378053

**Published:** 2017-10-13

**Authors:** Ursula M. Beer, Mark A. Neerincx, Nexhmedin Morina, Willem-Paul Brinkman

**Affiliations:** ^a^ Department of Intelligent Systems, Delft University of Technology, Delft, the Netherlands; ^b^ TNO Perceptual and Cognitive Systems, Soesterberg, the Netherlands; ^c^ Institute of Psychology, University of Münster, Münster, Germany

**Keywords:** Resilience training, reappraisal, virtual agent, interrupted time series, computer-based training, entrenamiento de resiliencia, reevaluación, agente virtual, series temporales interrumpidas, formación mediante, ordenadores, 心理韧性训练, 再评估, 虚拟代理, 中断时间序列, 电脑训练, • A multi-session computer-supported appraisal training is proposed.• The case study reveals the training successfully improves appraisals.• Post-training measures reveal more diverse and concise appraisals.• Negative side effects were not found.•There is a need for further investigation for long-term effects and including a control group.

## Abstract

**Background**: First responders are a prime example of professionals that are at a high risk of being exposed to traumatic experiences. Reappraisal as a coping strategy might help first responders to better cope with their emotional responses to traumatic events.

**Objective**: This study investigated the effects of repeated sessions of a digital reappraisal training among seven firefighters. The training consisted of four sessions supported by a virtual agent, conducted at home or at work, over a two-week period in a single case series.

**Method**: Sixteen data points were collected from each participant in the eight days pre- and post-training.

**Results**: Significantly more themes were used at post-training than at pre-training, implying more flexibility and confirming the main hypothesis of the study. Negative side effects were not reported during or in the week after the training.

**Conclusions**: More controlled studies into the short- and long-term effects of a training of this nature are needed. Furthermore, it provides a reference for developers in this field.

## Abstract (es)

**Planteamiento:** Los trabajadores de primeros auxilios son un buen ejemplo de profesionales con un alto riesgo de exponerse a experiencias traumáticas. La reevaluación como estrategia de afrontamiento podría ayudar a los trabajadores de primeros auxilios a lidiar mejor con sus respuestas emocionales a los acontecimientos traumáticos.

**Objetivo:** Este estudio investigó los efectos de sesiones repetidas de un entrenamiento de reevaluación digital en siete bomberos. La formación consistió en cuatro sesiones apoyadas por un agente virtual, realizadas en el hogar o en el trabajo, durante un período de dos semanas en una serie de casos únicos.

**Método:** Se recogieron dieciséis puntos de datos de cada participante en los ocho días previos y posteriores a la formación.

**Resultados:** Se utilizaron muchos más temas después de la formación que antes de la misma, lo que implica mayor flexibilidad y confirma la hipótesis principal del estudio. No se indicaron efectos secundarios negativos durante la formación ni en la semana posterior.

**Conclusiones:** Se necesitan más estudios controlados sobre los efectos a corto y largo plazo de una formación de esta naturaleza. Además, proporciona una referencia para los desarrolladores en este campo.

## Abstract (zh)

**背景：**急救人员是暴露于创伤体验中的高风险职业人群的典型例子。使用再评估作为策略可能帮助急救人员更好地应对他们对创伤事件的情绪反应。

**目的：**本研究考察了7个消防队员重复使用电子再评估训练的效果。训练包括由一个虚拟代理支持的四个部分，在家中或者工作中进行，每个个案序列持续两周。

**方法：**在8天训练中，每天训练前后对每个参加者总共收集了16个数据点。

**结果：**训练后比训练前明显有更多的主题被使用了，说明了更高的灵活度，同时也验证了研究的主要假设。在训练中和训练后一周内没有负面副作用被报告。

**结论：**需要进行更多关于相似训练的短期和长期效果的控制研究。同时，研究也为该领域开发者提供了参考。

## 1. Introduction and background

It is estimated that most people experience at least one potentially traumatic event during their lifetime (Kessler, Sonnega, Bromet, Hughes, & Nelson, 1995; Perkonigg, Kessler, Storz, & Wittchen, 2000). When untreated, post-traumatic stress disorder (PTSD) has a chronic course (Morina, Wicherts, Lobbrecht, & Priebe, 2014), is associated with significant mental and physical distress (Nemeroff et al., 2006) as well as high economic burden (Sabes-Figuera et al., 2012; Wittchen et al., 2011). First-responders (e.g. army, police, firefighters, etc.) are at a high risk of experiencing traumatic events and research indicates that the prevalence of PTSD among this population lies between 7.5 and 25% (Berger et al., 2012; Impact, 2008; Skogstad et al., 2013).

Determining why some individuals are more mentally resilient than others is a complex exploit, as depicted by the many definitions of the concept of resilience. It is, however, generally posed that resilience is a determinant of PTSD (Agaibi & Wilson, 2005; Marmar et al., 2006). Agaibi and Wilson’s (2005) review of resilience and trauma literature points to factors such as personality, prior traumatic experience, social support and coping styles as potential indicators of resilience. There are various coping styles one can implement (e.g. Aldwin & Yancura, 2004; Folkman & Moskowitz, 2000), and the preference and effectiveness of each differs per individual. This paper will focus on emotion regulation through positive reappraisal.

Skogstad et al. (2013) found indications that personnel trained to deal with stressful situations were at a lower risk of PTSD, thus a preventative training to prepare how to effectively cope with life stressors seems especially relevant for individuals in professions with a high risk of experiencing traumatic events. Several technology-supported resilience trainings have been investigated in previous studies, including predeployment stress inoculation training (Hourani, Kizakevich, & Hubal, 2011), stress resilience in virtual environments (Rizzo et al., 2011), immersion and practice of arousal control training (Bouchard, Bernier, Boivin, Morin, & Robillard, 2012) and the stress resilience training system (Cohn, Weltman, Ratwani, Chartrand, & McCraty, 2010) (for a review see Favié, Vakili, Brinkman, Morina, & Neerincx, 2016). However, many of these trainings focus on cognitions and behaviour during the traumatic experience, while less research attention has been directed at the cognitive processing after the event.

A powerful tool in coping with stress is to reinterpret the situation so as to regulate the emotions that are associated with it. The term positive reappraisal was coined by Lazarus and Folkman (1984) and is based on Lazarus’ notion that people are constantly appraising or evaluating situations on whether they are harmful (threat) or beneficial to their well-being. Lazarus proposed two phases of appraisal: primary and secondary. Primary appraisal is the constant assessment of whether a given situation represents a threat. Subsequently or simultaneously, secondary appraisal is an assessment of whether one is capable of dealing with the stressor, which can be positive or negative.

When there is enough time to re-evaluate appraisals of threat, we can reduce stress and emotional reactions by generating thoughts that are reassuring or by focusing on positive information (Lazarus, 1991; Mikulincer & Florian, 1996; Tugade & Fredrickson, 2004). Instead of reappraising a past event, appraisal modification has also been considered as a way of coping with new traumatic events. For example, Schartau, Dalgleish, and Dunn (2009), studied the effects of practicing appraisal themes using cognitive bias modification methodology. During the training, four appraisal themes (e.g. every cloud has a silver lining), were explained and participants practiced applying these themes to distressing films. In a series of four studies, the authors found a tendency towards reduced levels of electro dermal responses and self-reported negative emotional responses to a test film post-training, as well as fewer intrusions. The appraisal themes provided a framework to aid in assigning different views to a given situation.

Woud, Postma, Holmes, and Mackintosh (2013) found that positive reappraisal training prior to exposure to a film with traumatic content led to less reported distress arising from intrusive memories of the film in the week after in comparison to those who received negative reappraisal training. The authors concluded that a reappraisal training can be beneficial prior to a traumatic event. From another perspective, Bryant and Guthrie (2005) found that maladaptive appraisals can be a risk factor for posttraumatic stress among trainee firefighters.

Writing and reflecting about traumatic events can have various benefits (Pennebaker, 1997), including physical (King & Miner, 2000) and cognitive (Hemenover, 2003; Park & Blumberg, 2002) benefits. Building on these findings, we propose a reappraisal training that focuses on writing (i.e. reflecting on) positive appraisals in response to negative videos. Our training is supported by a virtual agent, the virtual coach, as well as a video labelling tool for aiding during the exercises. Digitizing the training makes this economical as well as easy to access. One can use the system at home or at work, and the content can be customized to the individual and/or to his or her profession (i.e. firefighters, soldiers, ambulance staff). A notable difference compared to reappraisal for therapeutic purposes is that for this training individuals write about events depicted in videos, rather than events they have experienced themselves in the past.

The training is based on Schartau et al.’s (2009) training in perspective broadening and is intended as a preventative measure to prepare individuals for future life stressors. It is a form of cognitive bias modification where the focus lies with practicing reappraisal in response to potentially distressing films. Participants are instructed via the Assisted Video Annotator system. This system includes a virtual coach and instructional videos about the four appraisal themes (positive thinking, bigger picture, personal growth, acceptance). Furthermore, a video annotation tool is embedded in the system and is used to practice applying the appraisal themes (see section 2.4. for more information).

The main hypothesis put forward is that engaging in repeated sessions of the technologically enhanced appraisal training will result in more positive appraisals in response to films depicting negative events. We assume that the improvement of appraisals is reflected through more flexibility in themes used, a larger amount of appraisals generated and more words written. Secondly, and in line with the cognitive model by Ehlers and Clark (2000), our second hypothesis estimates a shift in processing style from data-driven processing (associated with PTSD) to conceptual processing (more healthy). Furthermore, we expect that the training improves the individuals’ subjective ratings of skill and confidence. Finally, we wanted to examine potential negative side effects participating in such a training caused, for example by the nature of content of the training material.

## 2. Methods

### 2.1. Design

The case study had an interrupted time-series design, conducted among firefighters. This quasi-experimental design aims to determine whether there is a change in the outcome level or slope from baseline to post-intervention, i.e. whether this is an effect of the training on top of any possible natural development or attrition (Bernal, Cummins, & Gasparrini, 2016; Penfold & Zhang, 2013; Shadish, Kyse, & Rindskopf, 2013; Wagner, Soumerai, Zhang, & Ross‐Degnan, 2002). The study consisted of three phases: pre-training phase of eight days, a two-week intervention (training) phase, and an eight-day post-training phase. In the pre- and post-training phases, eight daily responses to films were collected, resulting in a total of 16 data points per participant.

### 2.2. Participants

Participants were recruited through posters and flyers posted at nine fire stations, as well as an e-mail invitation sent to all active firefighters within the organization. Of the 15 respondents, two withdrew participation due to insufficient time to commit to the study, and two did not respond after having signed up. The remaining respondents (*n *= 11) filled in the online informed consent form. By signing this form, participants indicated they did not suffer from uncorrected vision problems, autism spectrum disorders, emotional problems, anxiety and depression. One potential participant was excluded due to a recent traumatic experience. Following the consent form, participants completed the anxiety scale of the Hospital Anxiety and Depression Scale (HADS-A; Bjelland, Dahl, Haug, & Neckelmann, 2002), with the criterion that participants who score 8 or higher on this scale are excluded (*n *= 1). Finally, the Beck Depression Inventory-II (BDI-II; Beck, Steer, & Brown, 1996) was filled in with a cut-off of above 15 (none of the scores were above this cut-off). Ten respondents, all males, with a mean age of 36 years (SD = 10.8), participated in the study. Two of these dropped out during the pre-training phase and a further three dropped out during the training phase due to other commitments. Two of these did return their pre-training data. This made it possible to run an intention-to-treat analysis on the data of seven participants, even as no post-training measurement data were available for those two participants. Participants’ work experience ranged from 1 to 25 years, with an average of 149 accumulated negative events including exposure to fires, serious injuries, traffic accidents, witnessing death, abuse, aggression, etc. All returned data (*n *= 7) was used in the analyses.

### 2.3. Materials

#### 2.3.1. Appraisal themes

The training focused on practicing to look at situations from a different (positive) perspective. This was done with appraisal themes similar to those of Schartau et al. (2009). Upon consultation with a domain expert and a social worker at the fire department, the following four themes were chosen as most applicable to the population: seeing the positive aspects (silver lining), giving meaning (i.e. broader perspective), finding personal growth opportunities, and acceptance of the negative event.

#### 2.3.2. Films

The trauma film paradigm (Holmes & Bourne, 2008) has been shown to induce some symptoms reflective of PTSD: short-term physiological and psychological stress symptoms, as well as causing intrusive memories. However, the films in the current study, unlike other trauma film studies, were not chosen with the aim to incite stress reactions; rather, to challenge participants to think about and reflect on re-appraising the negative event. This aim is reflected in the first hypothesis of improving the flexibility in themes and number of appraisals one is able to construct.

A selection of 24 films was made containing coverage of distressing events that have been associated with the development of PTSD (de Vries & Olff, 2009; Wagner, McFee, & Martin, 2008), including six firefighter-specific videos, four other first responder-specific videos, as well as nine general PTSD-related videos (including but not limited to injury, violence, threat and natural disasters). Video material was gathered from news items and documentaries aired on Dutch television. The duration of the videos varied (circa 4–9 minutes). To make it easier to relate to the situations depicted in the videos, each video was introduced with a context/perspective which the viewer should keep in mind while watching. For example, when watching a news report about attacks during the Boston Marathon in 2013, participants were asked to view the videos from the perspective of an athlete that ran the marathon. All of the films and context were approved for acceptability and relevance by the social worker affiliated with the fire department as well as one of their in-house trainers.

### 2.4. System

Vakili, Brinkman, Morina, and Neerincx (2014) formulated several development guidelines and intervention requirements for developing a computer-supported resilience intervention for military personnel. Some of the proposed guidelines include consideration of the culture, effectiveness, engineering and resources as important factors in the development of novel interventions. Furthermore, technology is positively seen by soldier-trainees and stakeholders and it should be acknowledged that within the military there is some degree of stigma towards disclosing or discussing psychological problems. Training requirements include, among others, achieving behavioural change that enhances resilience, personalization to individual needs, durable and measurable effects, relevance to cultural context, and it should be economical, safe, engaging and motivating. These guidelines and requirements were key to the development of the proposed training. Several of these were addressed via the opportunity to follow the training individually, at home or at work in our study.

All digital materials (films and the training with virtual coach) were provided on a USB flash drive, accompanied by instructions and questionnaires. The training was built with the Assisted Video Annotator software, custom made by CleVR. The Assisted Video Annotator system encompasses the training, including the virtual coach and video annotation tool. Upon starting up the program, an overview of the sessions including a summary of each session was presented. Participants selected the session they were going to complete, which was done chronologically as skipping sessions was disabled. The entire session had to be completed before moving on to the next one.

**Figure 1.**
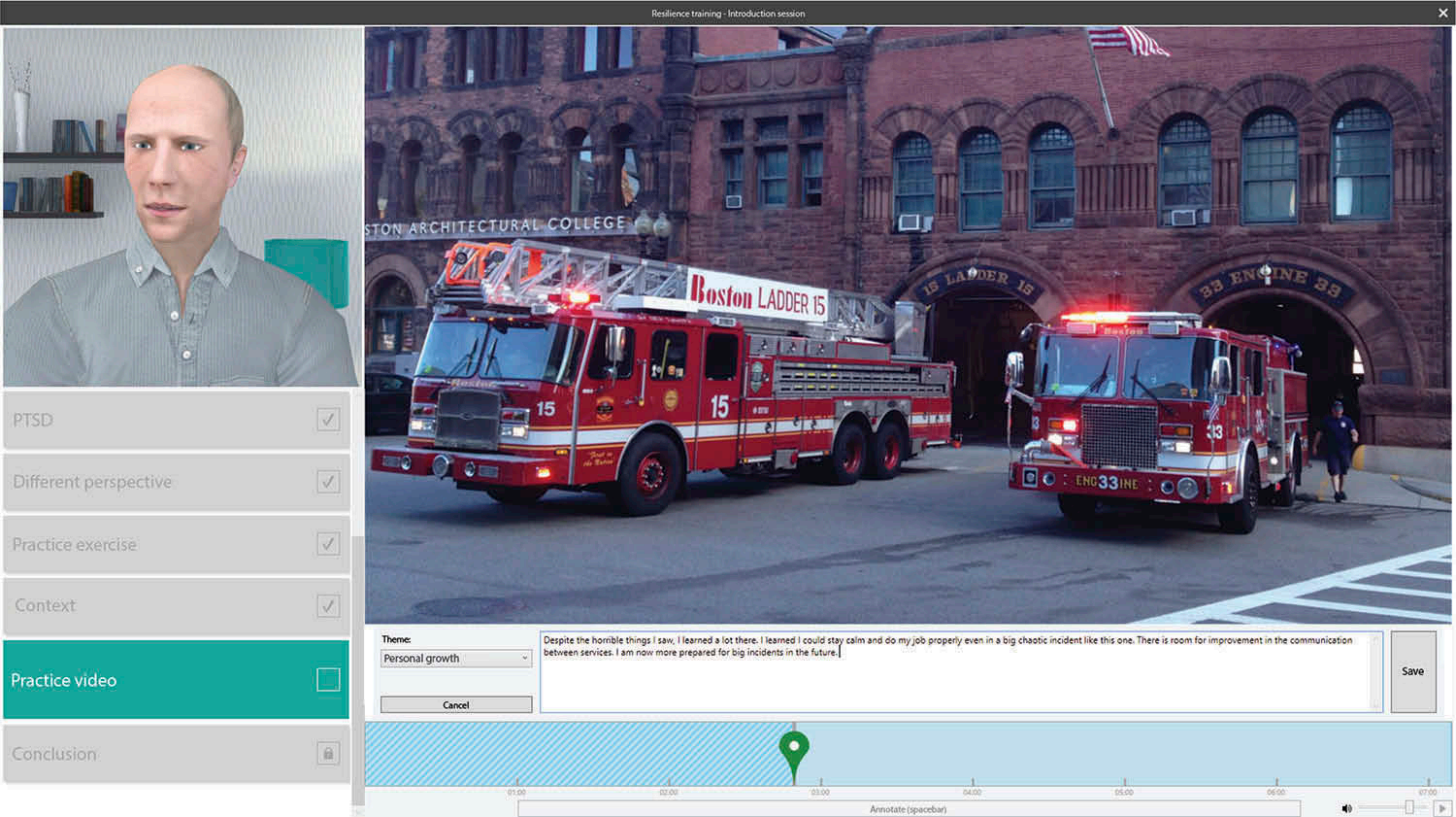
Screenshot of the labelling exercise within the training. For publication purposes the content has been edited and translated to English. Additionally, during the labelling exercise the virtual coach is blacked out so as not to make participants uncomfortable or distracted while watching the videos.

During the first session, the introduction session, participants were greeted by the virtual coach as he introduced himself. This was done through text-to-speech software. The virtual coach explained the purpose and goals of the training as well as discussing PTSD and the use of appraisal. During this session, an informative video was presented to explain the appraisal themes as well as a tutorial video on how to use the annotation tool. Participants practiced using the themes and the annotation tool. In the following three sessions participants used the labelling tool to apply the themes to three different videos per session. An example of such a labelling exercise is depicted in Figure 1.

Practice exercises involved using the annotation tool to label videos. The annotation tool is a video player that includes an interactive timeline on which participants can label instances during the video with a particular theme. The tool also allows the participants to reflect and expand (in writing) on the label they applied.

During the exercises, the virtual coach provided feedback based on three types of behaviour of the participant: too few labels, not enough text written and/or inactivity. Feedback included encouragement to create more labels, questions to help guide the participant to write more and reassurance upon inactivity. In each successive session, the virtual coach challenged the participants to create an additional label to the films. Also, the virtual coach provided less support in the form of examples towards the last session. The final session concluded with a recap of the topics covered during the training.

### 2.5. Primary outcome measures

#### 2.5.1. Appraisals

During the pre- and post-training measures, participants were asked to write freely (on paper) about which themes they applied as well as their thoughts and feelings about the video they had just watched on a regular video player. Two coders, namely the first author and a project-independent coder, counted the number of themes as well as the number of appraisals applied. A high degree of reliability was found between coders. For the number of themes, the average measure Intraclass Correlation Coefficient (ICC) was .87 with a confidence interval from .80 to .91 (*F*(93,93) = 7.5, *p *< .001). For the number of appraisals, the average measure ICC was .86 with a confidence interval from .79 to .91 (*F*(93,93) = 7.1, *p *< .001). The mean of the two coders’ scores were used for analysis. Counts of how many words participants had written in response to each film were also recorded.

#### 2.5.2. Processing style

The Cognitive Processing Questionnaire (CPQ; Halligan, Clark, & Ehlers, 2002) was administered as another indicator of resilience. With this scale, the data-driven and conceptual processing of traumatic material is measured. Ehlers and Clark’s cognitive model of PTSD (Ehlers & Clark, 2000) suggested that a greater use of data-driven processing (perception on a sensory level or characteristics of the traumatic event), rather than conceptual processing (processing the meaning of the event and its context) is associated with the development and maintenance of PTSD symptoms.

#### 2.5.3. Skill and confidence

To measure participants’ self-reported skills and confidence with positive reappraisal, six items were scored on a 100-point analogue scale. These items included: ‘If I were to experience this in real life I would handle it well’, ‘I can see [positive/negative] aspects in the situation’, ‘I find it difficult apply the themes’, ‘If I approach this from a healthy perspective (i.e. by applying the themes), I would assess the situation as [positive/negative]’.

### 2.6. Secondary outcome measures

#### 2.6.1. Affect

To examine the potential side effects of the training, the Positive and Negative Affect Schedule (PANAS; Watson, Clark, & Tellegen, 1988) was included. It measures 20 aspects of mood (10 positive and 10 negative adjectives) on a 5-point Likert-type scale. Directly after each video, participants were instructed to indicate the extent to which they felt this way during the video. Reappraisal is a form of emotion regulation, therefore a measure of subjective emotion in response to the videos was included. As the videos in this study were of a negative nature, yet not aimed at inciting horror and distress, a decrease in positive mood was to be expected after repeated viewing of such content.

#### 2.6.2. Intrusion diary

Potential side effects were further examined with an intrusion diary. All pre- and post-training measures (starting from the second) began with an intrusion diary in which participants report how many intrusions of film-related material they had since the last session, and on a scale of 0–100% how distressing, how vivid and what the content was (Lang, Moulds, & Holmes, 2009; Morina, Leibold, & Ehring, 2013). As frequent and prolonged intrusions are a classic symptom of PTSD, this measure was included in the study to determine whether the training might have any side effects on this phenomenon, due to the nature of the films.

#### 2.6.3. Film manipulation check

As a control measure, to determine whether participants were paying attention to the film, participants were asked whether they had seen it before and whether they understood the content (yes or no). They were also asked to what extent they could empathize with the film, whether they found the film distressing and three items on whether they were paying attention (on a 5-point Likert-type scale) (Morina et al., 2013).

### 2.7. Procedure

Once participants passed the online screening for trauma, anxiety and depression, they received (personally or by mail) a package containing a questionnaire on demographic information (age, gender, education) and personal experience with traumatic events. A handout with information on the appraisal themes (including examples to familiarize participants with the concept) was included to aid them in their reflections. Additionally, they received eight pre-training measures and a USB flash drive containing the eight corresponding films for the pre-training measures. Participants were instructed to complete one per day, preferably on successive days, however due to the unpredictability that accompanies their profession, leniency was tolerated.

**Figure 2.**
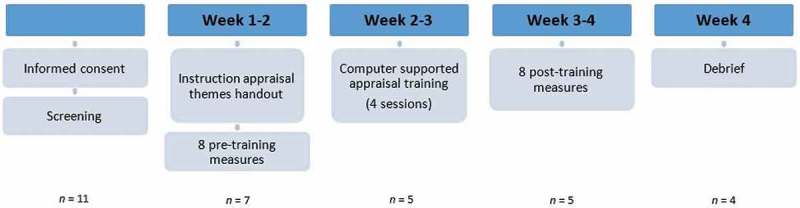
Overview of the procedure of the study.

After the pre-training measures, participants received instructions for installing the digital training and were requested to complete the four training sessions over a two-week period, with at least 1–2 days between sessions to allow for long-term consolidation (Denny & Ochsner, 2014). After having completed the training they received eight post-training measures and eight corresponding new films to complete on a daily basis. The order of the films was randomized (across pre- and post-training measures, as well as sessions 2–4 of the training) between participants. Finally, participants were invited to participate in a short debrief and interview (in person or via Skype) regarding the training as a whole. Participants were rewarded with a €20 gift voucher as a small token of appreciation for their participation. The procedure is summarized in Figure 2.

### 2.8. Data preparation and analysis

Data was analysed using R version 3.2.4 (R Core Team, 2016). Of the six items regarding skill and confidence, one item was dropped – ‘I can see the negative aspects of the situation’ – as it had a low correlation with other items. This item was problematic because it did not measure the desired skills the training aims to improve. The mean scores of the five remaining items were calculated to create a single Skill and Confidence score (α = 0.85).

The first step was to statistically test the effects of the training on the outcome variables, thus we fit multilevel models to the data (Shadish et al., 2013). The variable Measure number was created, which is merely the general progression of the 16 measurement moments. Note that participants started on different dates and had varying gaps between measures, hence participants were treated as a random intercept in all multi-level models. Furthermore, the Condition variable was used to determine the effect of the training, i.e. whether it was a pre- or post-training measure. A null-model (Model 0), formally written as *y_ij_* = γ_00_ + *U*_0*j*_ +ε_ij_ was created. This model was then expanded by adding the fixed effect of Measure number (Model 1) and Condition (Model 2) to determine a change in level. Finally, to examine a potential change in slope, the interaction of Measure number and Condition was added to create Model 3. As no significant interaction effect in Model 3 was found, nor an improvement of the model’s fit, only Model 2 is reported in the results section.

**Figure A1.**
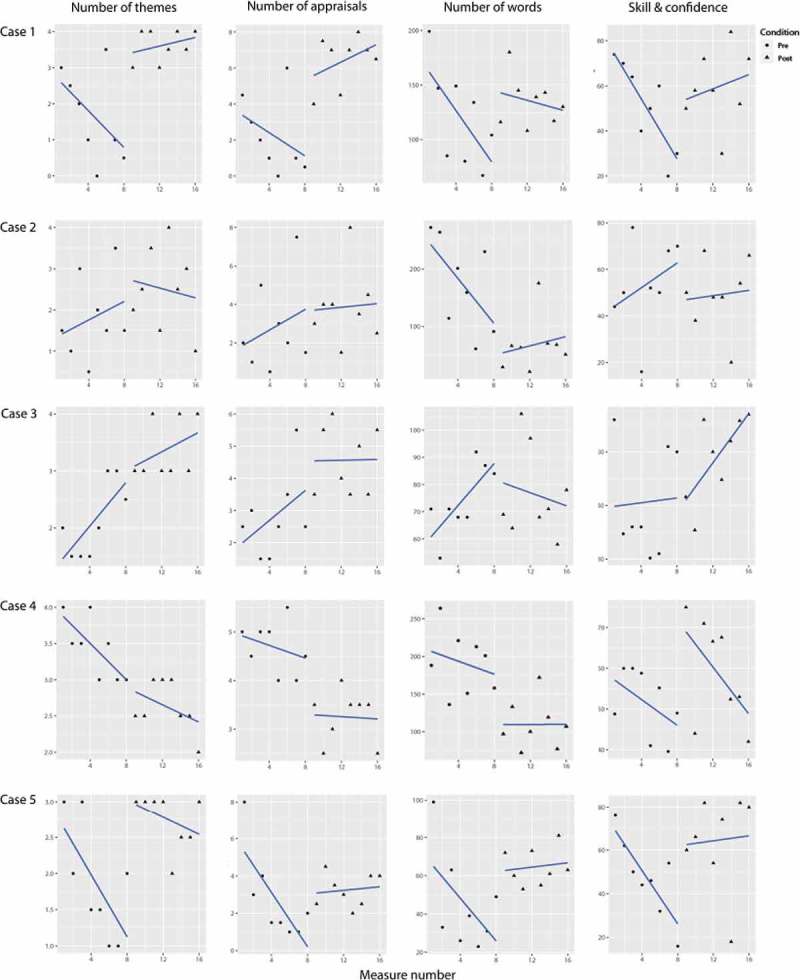
Data of primary outcome measures for individual cases of completers.

After aggregated analysis, individual progress was examined visually (see figure A1 of the Appendix for further information).

## 3. Results

### 3.1. Film manipulation check

The questions regarding the film manipulation check revealed that all participants understood what the film was about. Of the aggregate responses, 46.7% of the video content had been seen prior to the study, 53.3% had not. Participants were empathetic with regard to the people in the films (*M *= 4.28, *SD* = 0.87, range = 1–5) and reported moderate distress when watching the films (*M *= 3.24, *SD* = 1.17, range = 1–5). Furthermore, participants paid attention to the films (*M *= 4.05, *SD* = 1, range = 1–5), reported no distraction while watching the films (*M *= 1.4, *SD* = 0.73, range = 1–5) and did not avert or close their eyes while watching the film (*M *= 1.13, *SD* = 0.4, range = 1–3).

### 3.2. Primary outcome measures

The results of the multi-level analysis of all primary outcome measures are provided in Table 1 and Table 2.

**Table 1.** Multilevel analyses results of primary outcome measures: number of themes, number of appraisals and word count.

	Number of themes				Number of appraisals				Word count			
Model 2	*B*	*SE*	*df*	*p*	*B*	*SE*	*df*	*p*	*B*	*SE*	*df*	*p*
Intercept	2.35	0.28			3.29	0.52			136.51	19.78		
Measure number	−0.03	0.04	86	0.48	−0.04	0.09	86	0.62	−4.68*	2.24	87	0.04
Condition	0.99*	0.39	86	0.01	1.51^†^	0.81	86	0.07	5.58	20.9	87	0.79
	*χ^2^(1)*	*p*			*χ^2^(1)*	*p*			*χ^2^(1)*	*p*		
Model 0 vs. 1	4.37*	0.04			2.65	0.1			8.87**	<0.01		
Model 1 vs. 2	6.25*	0.01			3.43	0.06			0.07	0.79		

^†^ *p* < .1, *p < .05, **p < .01.

**Table 2.** Multilevel analyses results of primary outcome measures: Processing style and Skill and Confidence scores.

	Conceptual processing				Data-driven processing				Skill and Confidence			
Model 2	*B*	*SE*	*df*	*p*	*B*	*SE*	*df*	*p*	*B*	*SE*	*df*	*p*
Intercept	13.97	1.1			8.17	1.48			56.33	6.91		
Measure number	−0.15	0.1	87	0.12	0.2	0.19	87	0.31	−0.92	0.65	87	0.16
Condition	1.52	0.92	87	0.1	−2.52	1.81	87	0.17	14.44*	6.14	87	0.02
	*χ^2^(1)*	*p*			*χ^2^(1)*	*p*			*χ^2^(1)*	*p*		
Model 0 vs. 1	0.07	0.79			0.06	0.8			0.98	0.32		
Model 1 vs. 2	2.7	0.1			1.82	0.17			5.3*	0.02		

^†^ *p* < .1, *p < .05, **p < .01.

#### 3.2.1. Number of themes

**Figure 3.**
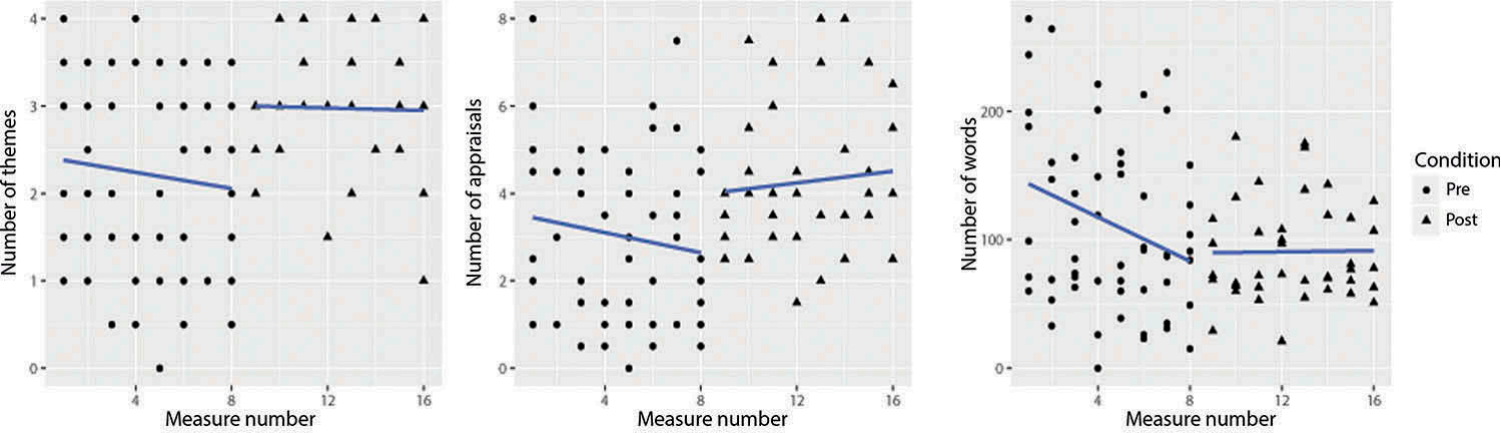
Aggregated data of all participants for number of themes, number of appraisals and number of words written.

The addition of Measure number to the null-model improved the model’s fit. Adding the condition (pre-/post-training) to create Model 2 also improved the model’s fit. Examination of the individual factors in Model 2 shows, however, only Condition as a significant fixed factor. Participants used on average one theme more at post-training. Participants’ scores on the pre- and post-training primary outcome measures are displayed in Figure 3.

#### 3.2.2. Number of appraisals

Adding Measure number to the null-model showed no significant improvements in the model’s fit regarding the number of appraisals written. The addition of Condition in Model 2 shows that there is a marginally significant average in the number of appraisals at post-training.

#### 3.2.3. Word count

The addition of Measure number improved the model’s fit, however adding condition to that model did not. Condition did not have a significant effect on the number of words written. A significant effect of the measure number on the number of words written in the reflections was found. Examining the word count graph in Figure 3, an initial decrease in the number of words with a stabilization at post-training is depicted.

#### 3.2.4. Processing style and Skill and Confidence

**Figure 4.**
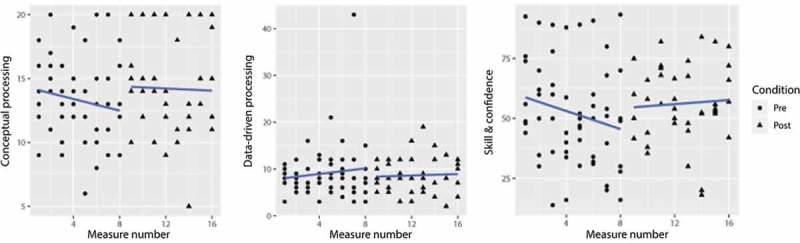
Aggregated data of all participants for processing style and Skill and Confidence scores.

Table 2 reveals no significant changes in the models’ fit for conceptual and data-driven processing scales. Table 2 does reveal an improvement in model fit on the Skill and Confidence measure. Examining the factors in Model 2, it showed that the model improvement could be explained solely by Condition. As depicted in Figure 4, there is a significant increase in perceived skills and confidence at post-training (*B* = 7.03, *SE* = 3.31, *p* = 0.04). Figure 4 provides a graphical representation of these factors.

#### 3.2.5. Individual examination

Examining the primary outcome measures on an individual level (see figure 1 of the Appendix) revealed that the aggregated findings could consistently be found back in the outcomes of four out of the five participants. Contra to other participants, in case four the number of themes and appraisals decreases after the intervention. A potential explanation is that this particular participant had the most work experience among all completers, with 25 years of work experience, whereas the other participants had 11 years or less of work experience. Furthermore, notes from the debriefing showed that the participant indicated that he had often thought back about his own similar experiences during the training and that this sometimes kept him up at night. He did not, however, report any intrusions.

### 3.3. Secondary outcome measures

#### 3.3.1. Affect

**Figure 5.**
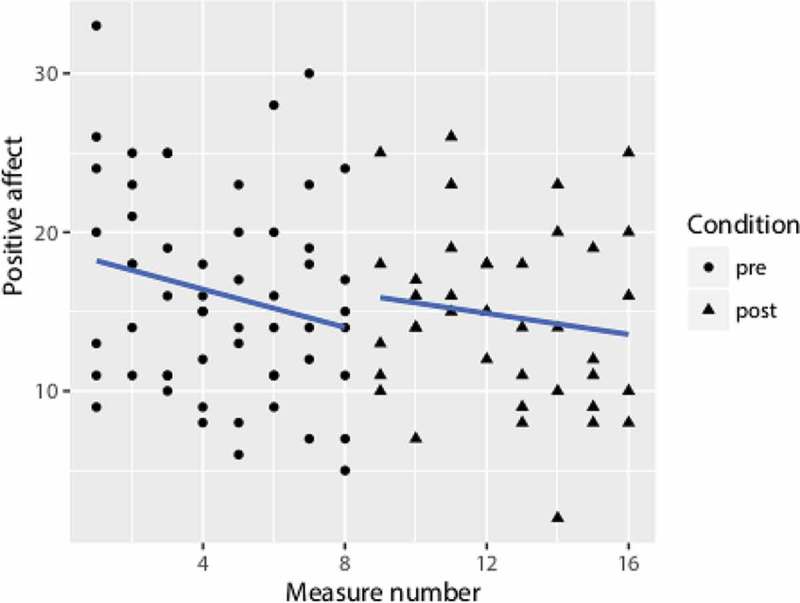
Aggregated data of Positive affect.

Table 3 reveals a marginally significant improvement in model fit as a result of adding condition. Table 3 also reveals, when considering the fixed factors in Model 2, Measure number has a significant effect and Condition only approached a significant level of .05. Figure 5 shows that, with the passing of time, positive affect decreases, with only what appears to be a slight upward shift at post-training. Negative affect did not have any significant change for Measure number nor Condition.

**Table 3.** Multilevel analyses results of secondary outcome measures.

	Positive affect				Negative affect			
Model 2	*B*	*SE*	*df*	*p*	*B*	*SE*	*df*	*p*
Intercept	18.33	1.82			8.88	1.75		
Measure number	−0.49*	0.22	87	0.03	0.03	0.25	87	0.91
Condition	3.75^†^	2.12	87	0.08	0.31	2.29	87	0.89
	*χ^2^(1)*	*p*			*χ^2^(1)*	*p*		
Model 0 vs. 1	1.62	0.2			0.16	0.7		
Model 1 vs. 2	3.13^†^	0.08			0.02	0.9		

^†^ *p* < .1, *p < .05.

#### 3.3.2. Intrusion diary

Cases one, three and five reported intrusions in their intrusion diary. However, during the debrief it became apparent that the participants had interpreted the definition of an intrusion in different ways (even though a definition was given). For example, case five revealed that he had reported remembering or thinking of the content in general. Others had written about their intrusions in a reflective manner, indicating some form of remembering that is not necessarily an intrusive memory. None of the intrusions scored higher than 30% on the realness and distress scales (0% being not at all real/distressing and 100% being very real/distressing).

## 4. Discussion

### 4.1. Main findings

Schartau et al. (2009) studied the effects of reappraisal training in the short-term, focusing on emotional recovery and change in thinking, whereas in this study we looked at appraisals and thought process over a longer period of time. The hypothesis that participants would be more flexible in applying appraisals following the four-session training was confirmed in that the number of themes and appraisals increased after the training. This can be understood as a more flexible construction of appraisals, since an increased use of themes was reported. Appraisal flexibility is important with regard to resilience as it harbours feelings of ‘mastery, competence, commitment, and other aspects of positive self-perceptions that maintain or restore self-esteem’ (Westphal & Bonanno, 2007). The intervention had no significant effects on the number of words. From the graphs we infer that the number of words decreased, but stabilized post-training. Taking this into account, along with the increased number of appraisals and themes, perhaps participants’ appraisal formation skills became more concise. This was further supported by the subjective skill and confidence measures, which increased at post-training.

The stability of the processing styles throughout the study could be due to the timing of this scale, as Halligan et al. (2002) put forward in their discussion. They posed that the effects of the processing style may be prolonged and better measured after long-term memory consolidation.

One very important issue when designing a training that can be done independently at home relates to its safety. During this study, participants did not report intrusions or an upward shift in positive affect after the training, which we interpreted as an indication of lack of negative side effects. A decrease in positive affect over time was to be expected, however this effect was marginally less so at post-training. These results differ from those of Schartau et al. (2009), and this could be due to the nature of the films, the effects of repeated exposure to negative content or perhaps participants found the task itself not engaging. No change in negative mood was reported. Films that depict horror can presumably have direct effects on emotions, however this effect was not found in our study.

### 4.2. Limitations

There are several limitations to this study that mostly result to the design of the study, i.e. a case study. Only a small number of firefighters were included in the study and thus a very specific group of professionals, limiting the generalizability of our results. Furthermore, there was no control group, but this was offset by including a baseline pre-training ‘control’ condition.

The number of pre- and post-treatment measures was relatively high and could have caused side-effects such as fatigue and habituation. Furthermore, these measures included watching a film and reflecting about it – which is very similar to the actual training exercises. One could posture that these measures should be considered as a part of the training in themselves, yet in the analysis we see a clear break in the trend at post-training.

The stimulus materials, i.e. the films, were not validated prior to the study. This was due to the nature of the application to be built to have content in line with the target population. Validated material is available, but mostly as static images or not domain specific. The number of validated films available (Carvalho, Leite, Galdo-Álvarez, & Gonçalves, 2012; Weidmann, Conradi, Gröger, Fehm, & Fydrich, 2009) is very limited, i.e. not enough for our purposes, and the length of these films and the topics they cover are not applicable to the fire fighters. Additionally, due to ethical considerations we chose not to repeatedly expose participants to horror films.

### 4.3. Future work

For continuation of this research we propose including a control group and a larger sample size. Additionally, this study could be broadened to include different professions and levels of experience (i.e. novice vs. experienced). Furthermore, we advise testing the dosage of the training sessions, perhaps providing more sessions but with a lower frequency of pre- and post-training measurements.

Our study cannot answer the question whether the appraisal skills are long-lasting. Long-term follow-ups could provide valuable insight into the long-term effects of such a training. We do not know if it contributes in the long term or whether it ensures ‘better’ cognitions. To illustrate this point, for many years (and wars) it was thought that psychological debriefing directly after a traumatic event was the best way to prevent PTSD, yet later research showed that this was not the case – it actually increased the chances of developing PTSD (Van Emmerik, Kamphuis, Hulsbosch, & Emmelkamp, 2002). However, despite that we cannot predict the long-term effects of our preventative training at this point, a case can be made that reappraisal skills contribute positively in the management of PTSD symptoms (Woud et al., 2013).

Technologically, a future system would provide feedback about what others would do in the presented situations, and it would be more active in providing examples during the practice exercises. With regard to the content, this training focussed on classical cognitive behavioural therapy theory, specifically emotion regulation through reappraisal. However, the training could be extended to include more elements such as other coping strategies and emotion regulation techniques (Aldwin & Yancura, 2004; Boden et al., 2013; Folkman & Moskowitz, 2000; Gross, 2002) to improve resilience on a broader scale.

This study has offered some insightful first results on the potential efficacy of a computer supported appraisal training. Future research needs to replicate the results in larger samples.
